# High expression of WNT7A predicts poor prognosis and promote tumor metastasis in pancreatic ductal adenocarcinoma

**DOI:** 10.1038/s41598-018-34094-3

**Published:** 2018-10-25

**Authors:** Dong-jin Wu, Yong-sheng Jiang, Rui-zhe He, Ling-ye Tao, Min-wei Yang, Xue-liang Fu, Jiang-yu Yang, Kun Zhu

**Affiliations:** 1 Department of Surgery, Shanghai Jiading Central Hospital, Shanghai, 201800 P. R. China; 2 Department of Biliary-Pancreatic Surgery, Ren Ji Hospital, School of Medicine, Shanghai Jiao Tong University, 1630 Dongfang Road, Shanghai, 200127 P. R. China; 3 Department of General Surgery, The people’s hospital of Suzhou National New &Hi-Tec Industrial development Zone, Suzhou, 215129 Jiangsu P. R. China

## Abstract

Due to the therapy resistance and frequent metastasis, pancreatic ductal adenocarcinoma(PDAC) remains one of the most malignant carcinoma. WNT7A, an important ligand of Wnt/β-catenin signaling pathways, has a controversial role in tumor development. The role of WNT7A in PDAC remains unclear. In this study, we analyzed the expression pattern of WNT7A at mRNA and protein levels. We found pancreatic cancer tissue demonstrated a significant high WNT7A expression compared with the adjacent non-tumor tissue and the expression of WNT7A positively correlates with poor prognosis and lymph node metastasis. Then, we performed transwell assays and wound healing assays *in vitro* and found that WNT7A promotes the migration capacity of cancer cells. Furthermore, we explored the underlying mechanism of the WNT7A inducing cell migration. Results showed that up-regulated WNT7A expression inducing higher expression of N-cadherin and lower expression of E-cadherin while the contrast result was shown in the WNT7A knock-down group, which suggested that WNT7A might contribute to an epithelial–mesenchymal transition. Finally, we found that the hypoxia culture condition remarkably increased the WNT7A expression. In conclusion, our work demonstrated that hypoxia induced high expression of WNT7A might promote the cell migration via enhancing the epithelial–mesenchymal transition in PDAC.

## Introduction

Pancreatic ductal adenocarcinoma remains one of the most lethal malignancies with frequent metastasis and recurrence^1^. Despite the relative low incidence, PDAC is the fourth cause of cancer-related deaths in developed countries with a 5-year survival rate of only 8.2%^2^. Though great effort has been made, little progression has been achieved in the diagnosis and treatment of PDAC. The survival rate for PDAC patients stays almost unchanged for more than 40 years^3^. Thus, a thorough knowledge for the underlying molecular mechanism is urgently needed to conquer this international challenge.

One of the main reasons causing extremely low survival rate of PDAC is that most patients are diagnosed with metastasis and are ineligible for curative surgical resection. Metastasis occurs due to the loss of cell to cell adhesion ability. Epithelial–mesenchymal transition(EMT) is a process in which epithelial cells acquire the mesenchymal phenotype, which has motile and invasive characteristics and easily to metastasis^4^. Over the years evidence is accumulated indicate that EMT is a vital step for malignance related metastasis^5,6^. Various Pathways regulating EMT including HGF, EGF,TGF-β, Notch, and Wnt/β-catenin signaling pathways^5^. Among them, Wnt/β-catenin pathway is of great importance. WNT genes family encode secreted proteins, acting as ligand to activate the Wnt/β-catenin pathway^7–9^. By different pathways they work through, the ligands are typically classified into canonical and non-canonical pathway^10,11^. As a member of Wnt family, WNT7A has been investigated in several types of cancer including ovarian cancer, cervical cancer, endometrial cancer, lung cancer etc.^12–14^. But there is conflicting evidence as to whether the role of WNT7A is tumor-promoting or tumor-suppressing. And the role of WNT7A in Pancreatic ductal adenocarcinoma remains unclear.

Here, we present the expression pattern and clinical significance of WNT7A in PDAC. And then we investigated the roles of WNT7A in PDAC and explore the possible mechanism involved in WNT7A-mediated functions.

## Results

### The expression of WNT7A significantly increased in PDAC at both mRNA and protein levels

**Figure 1:**
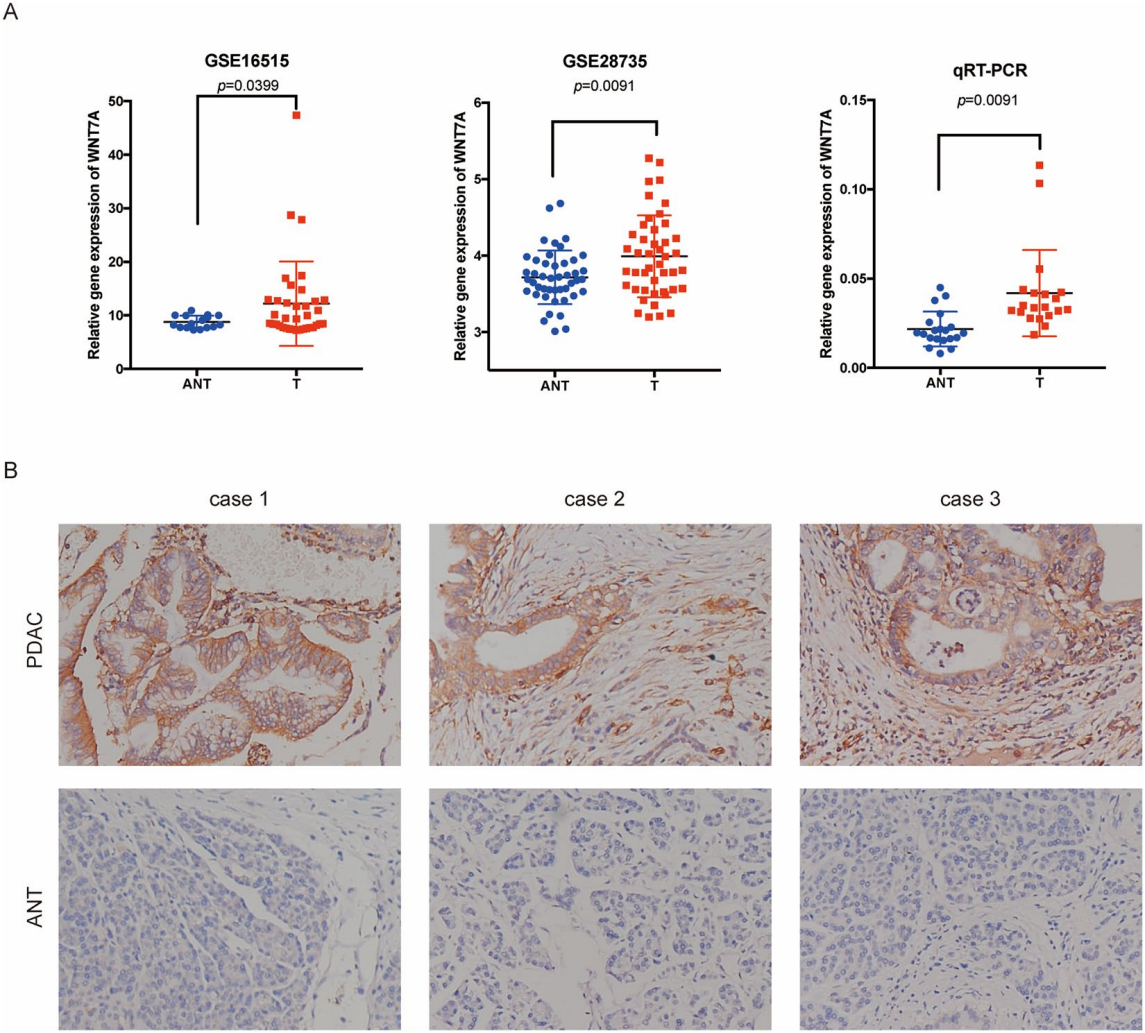
The expression of WNT7A significantly increased in PDAC at both mRNA and protein levels in datasets and TMA. (**A**) The expression of WNT7A at mRNA level in PDAC tissue and paired adjacent non-tumor tissue in GSE16515, GSE28735 and Ren Ji cohort. (**B**) The typical IHC staining of WNT7A in PDAC tissue and paired adjacent non-tumor tissue from the TMA.

To explore the expression pattern of WNT7A in PDAC, we re-analyzed the expression of WNT7A in two independent GEO datasets. We found that WNT7A expression dramatically increased in PDAC tissue compared with paired adjacent non-tumor tissue in GSE16515(*p* = 0.0399) and GSE28735(*p* = 0.0091). To verify the result, we also performed qRT-PCR to evaluate the WNT7A expression in 20 pairs of PDAC and adjacent-non tumor tissue from Ren Ji cohort. Data shows in Fig. 1A. The results suggested that WNT7A is remarkably increased in PDAC tissue compared to adjacent non-tumor tissue (*p* = 0.0091).

Furthermore, we evaluated the expression of WNT7A at protein level using PDAC tissue microarrays (TMA) which containing a cohort of 80 paired PDAC and normal pancreas tissue and another 20 non-paired tumor tissue. As shown in Fig. 1B, IHC staining of WNT7A specially located in the ductal like cells. The expression of WNT7A at protein level was evaluated based on both staining intensity and area, we found that 64 of 100 (64%) PDAC tissue with high expression of WNT7A while only 25 of 80 (31.25%) normal pancreas showed high expression of WNT7A (*p* < 0.0001). The classification criteria was explained in Method.

### Correlation of WNT7A expression and clinicopathologic features of PDAC

We performed *Chi-square* analysis to evaluate the relationship between WNT7A expressions and the clinicopathologic features of PDAC in TMA cohort. The clinicopathologic features including age, gender, tumor location, size, tumor differentiation, T classification, N classification, M classification, AJCC stage and neural invasion. The patients in TMA cohort have an average age of 61.66 years (from 34 to 85 years), 63 patients were males and 37 patients were females. As shown in Table 1, high expression of WNT7A significantly correlated with N classification (*p* = 0.033), which suggested a potential link between WNT7A and lymph node metastasis.

**Table 1:** Correlations of WNT7A with clinical characteristics in PDAC patients.

Characteristics	WNT7A Expression			
	Total	High	Low	P value
	(n = 100)	(n = 64)	(n = 36)	(χ^2^ test)
Age(years)				1.000
<60	47	30	17	
≥60	53	34	19	
Gender				0.426
Male	63	42	21	
Female	37	22	15	
Tumor location				1.000
Head	60	38	22	
Body/tail	40	26	14	
Size				0.541
≤3 cm	15	9	6	
>3 cm	83	53	30	
Tumor differentiation				1.000
Well	69	44	25	
Moderate/poor	31	20	11	
T classification				0.531
T1/2	78	50	28	
T3/4	20	12	8	
N classification				**0.033**
Absent	55	29	26	
Present	39	30	9	
M classification				0.535
Absent	98	62	36	
Present	2	2	0	
AJCC stage				0. 535
Stage I/II	98	62	36	
Stage III/IV	2	2	0	
Neural invasion				0.341
Absent	58	34	24	
Present	40	29	11	

AJCC staging is according to the 7^th^ edition of the American Joint Committee on Cancer (AJCC) staging system. The bold number represents the p-values with significant differences. P value was calculated by χ^2^ test or Fisher’s exact test.

### High expression of WNT7A predicts poor prognosis of PDAC patients

**Figure 2:**
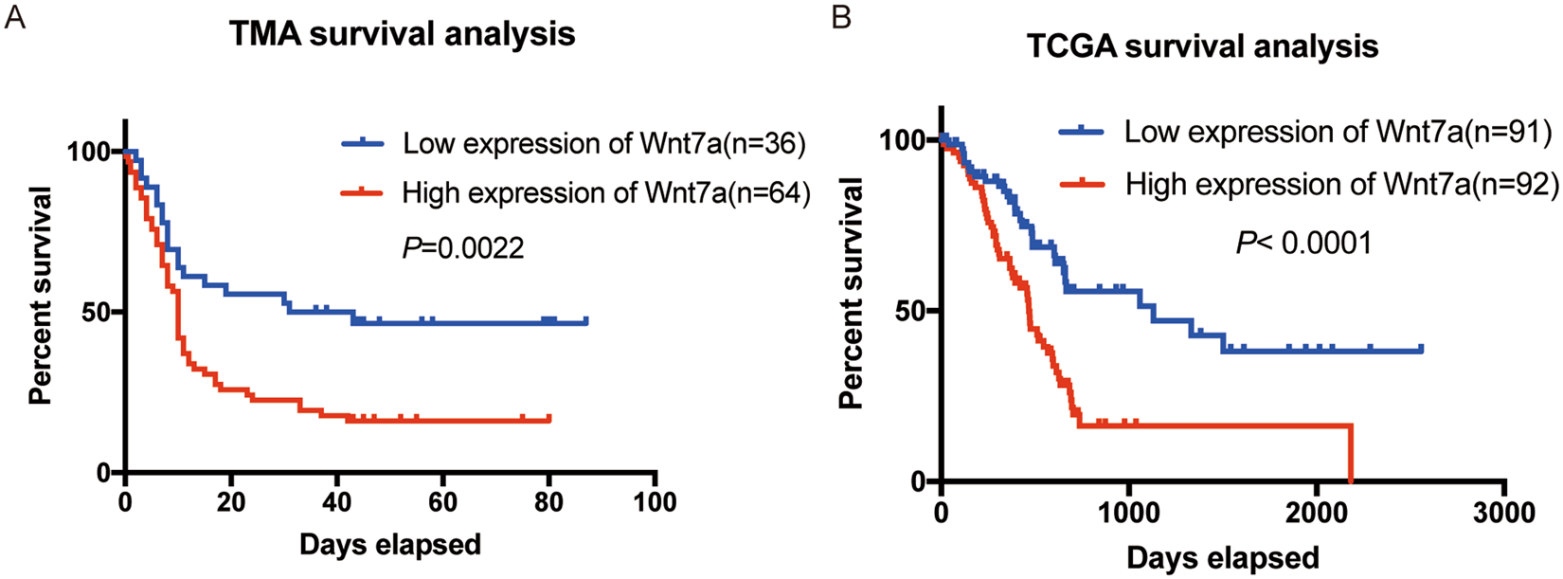
Kaplan-Meier survival analysis of WNT7A expression in PDAC patients. (**A**,**B**) Kaplan-Meier survival analysis based on the expression of WNT7A in TMA cohort (**A**) and TCGA cohort (**B**).

To assess the prognosis value of WNT7A in PDAC, we used Kaplan-Meier analysis and log-rank test to analyze the relationship between WNT7A expression and patients’ survival data. We found that patients with higher expression of WNT7A lived significantly shorter than those with a relative lower WNT7A expression (Fig. 2A, *p* = 0.0022). Furthermore, to verify the prognosis value of WNT7A, we also performed the Kaplan-Meier survival analysis in TCGA cohort which contained 183 PDAC patients. We divided those patients into two group according to the mRNA expression of WNT7A by dichotomic method. The results confirmed that patients with high expression of WNT7A lived dramatically shorter than those with low WNT7A expression (Fig. 2B, *p* < 0.0001).

We also used univariate and multivariate analysis to identify the risk factors for PDAC in TMA cohort. Univariate Cox regression analysis suggested that WNT7A expression, tumor differentiation as well as lymph node metastasis associated with poor prognosis. Then, multivariate Cox regression analysis demonstrated that WNT7A expression, tumor differentiation as well as lymph node metastasis were independent risks factors for PDAC patients (Table 2). Those results suggest that WNT7A is an independent risk factor for PDAC patients and might work as an oncogene in tumor progression.

**Table 2:** Univariate and multivariate analyses of prognosis factors for survival in PDAC patients. HR: Hazard ratio; CI: confidence interval. The bold number represents the p-values with significant difference.

Prognostic parameter	Univariate analysis			Multivariate analysis		
	HR	95%CI	P value	HR	95% CI	P value
WNT7A (high vs. low)	2.258	1.333–3.824	**0.002**	1.973	1.127–3.453	**0.017**
Age (≥60 vs.<60)	1.238	0.781–1.962	0.364			
Gender (male vs. female)	1.099	0.863–1.400	0.445			
Tumor location (head vs. body/tail)	0.946	0.750–1.194	0.641			
Size (>3 cm vs. ≤3 cm)	1.387	0.759–2.535	0.288			
Tumor differentiation (moderate/poor vs. well)	1.865	1.151–3.021	**0.011**	2.649	1.542–4.551	**0.000**
T classification (T3/T4 vs. T1/T2)	1.018	0.760–1.364	0.905			
N classification (present vs. absent)	2.119	1.305–3.440	**0.002**	2.322	1.359–3.967	**0.002**
M classification (present vs. absent)	1.757	0.429–7.189	0.443			
AJCC stage (III/IV vs. I/II)	1.757	0.429–7.189	0.433			
Neural invasion (present vs. absent)	0.909	0.719–1.150	0.427			

### WNT7A promotes the migration capacity of pancreatic cancer cells

**Figure 3:**
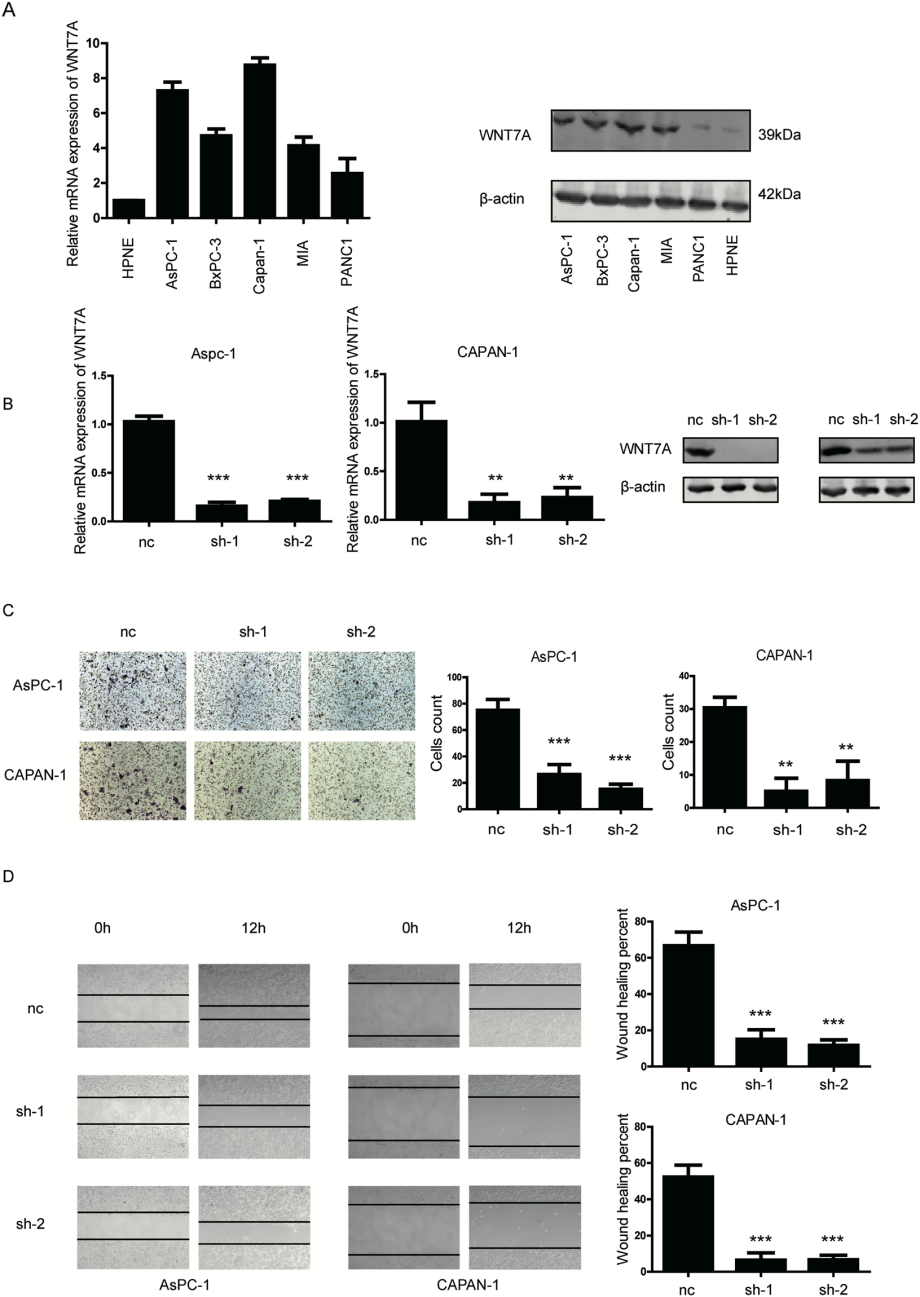
WNT7A is overexpressed in PDAC cell lines and interference of WNT7A expression inhibits the migration capacity of pancreatic cancer cells. (**A**) The mRNA and protein levels of WNT7A were detected in five different PDAC cell lines and a nonmalignant cell line hTERT-HPNE by qRT-PCR and Western blot respectively. (**B**) The expression of WNT7A at mRNA and protein level were determined by qRT-PCR and Western blot respectively in cells transfected with negative control and wnt7a shRNA plasmid. (**C**) Impact of WNT7A expression interference on two PDAC cell lines’ migration capacity in transwell assays. (**D**) Impact of WNT7A expression interference on two PDAC cell in wound healing assays. ^**^P < 0.01, ^***^P < 0.001.

**Figure 4:**
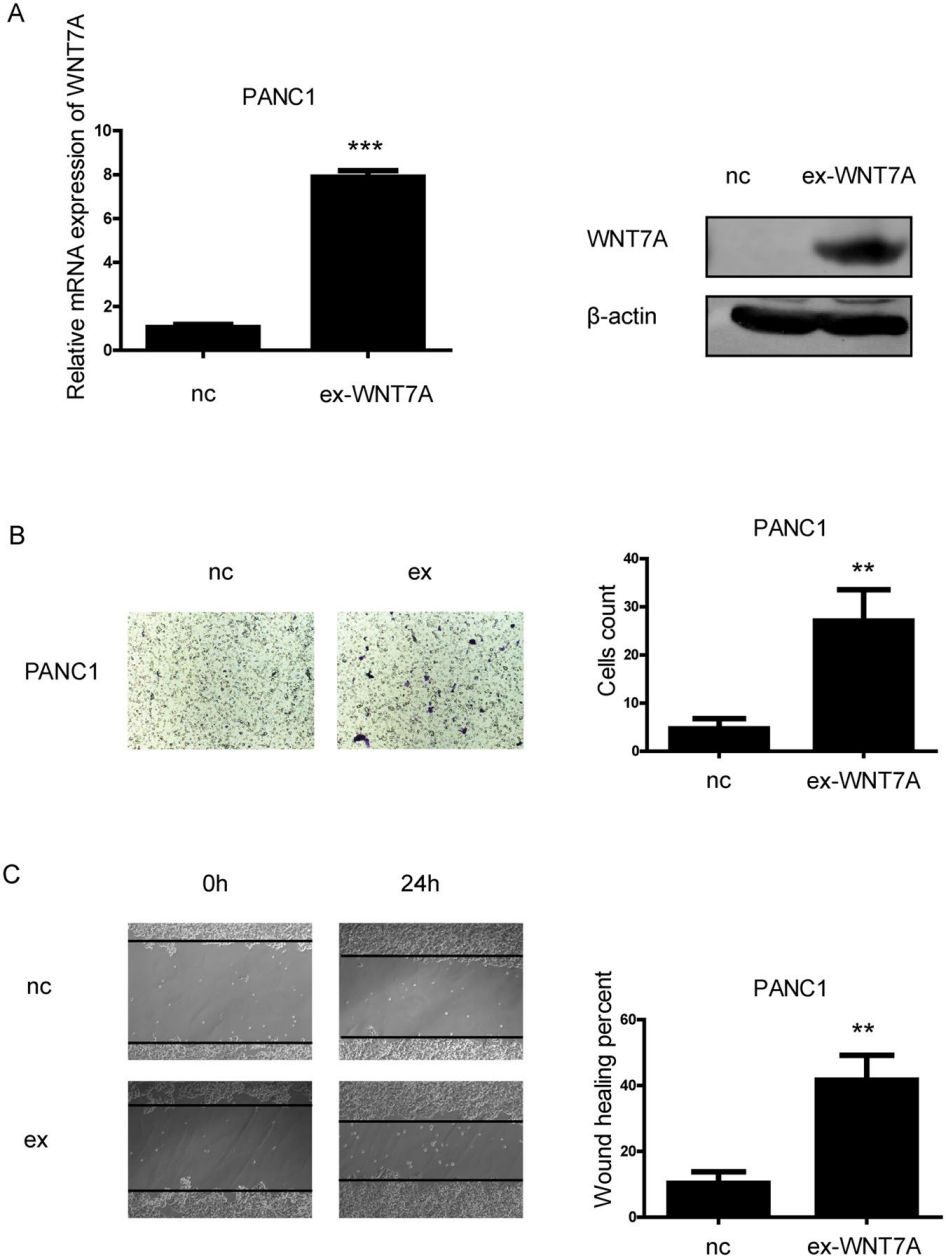
Over-expression of WNT7A promotes the migration capacity of pancreatic cancer cells *in vitro*. (**A**) The expression of WNT7A at mRNA and protein level were determined by qRT-PCR and Western blot respectively in cells transfected with negative control and wnt7a plasmid. (**B**) Impact of over-expression of WNT7A on PDAC cell’s migration capacity in transwells assays. (**C**) Impact of over-expression of WNT7A on PDAC cell in wound healing assays. ^**^P < 0.01, ^***^P < 0.001.

**Figure 5:**
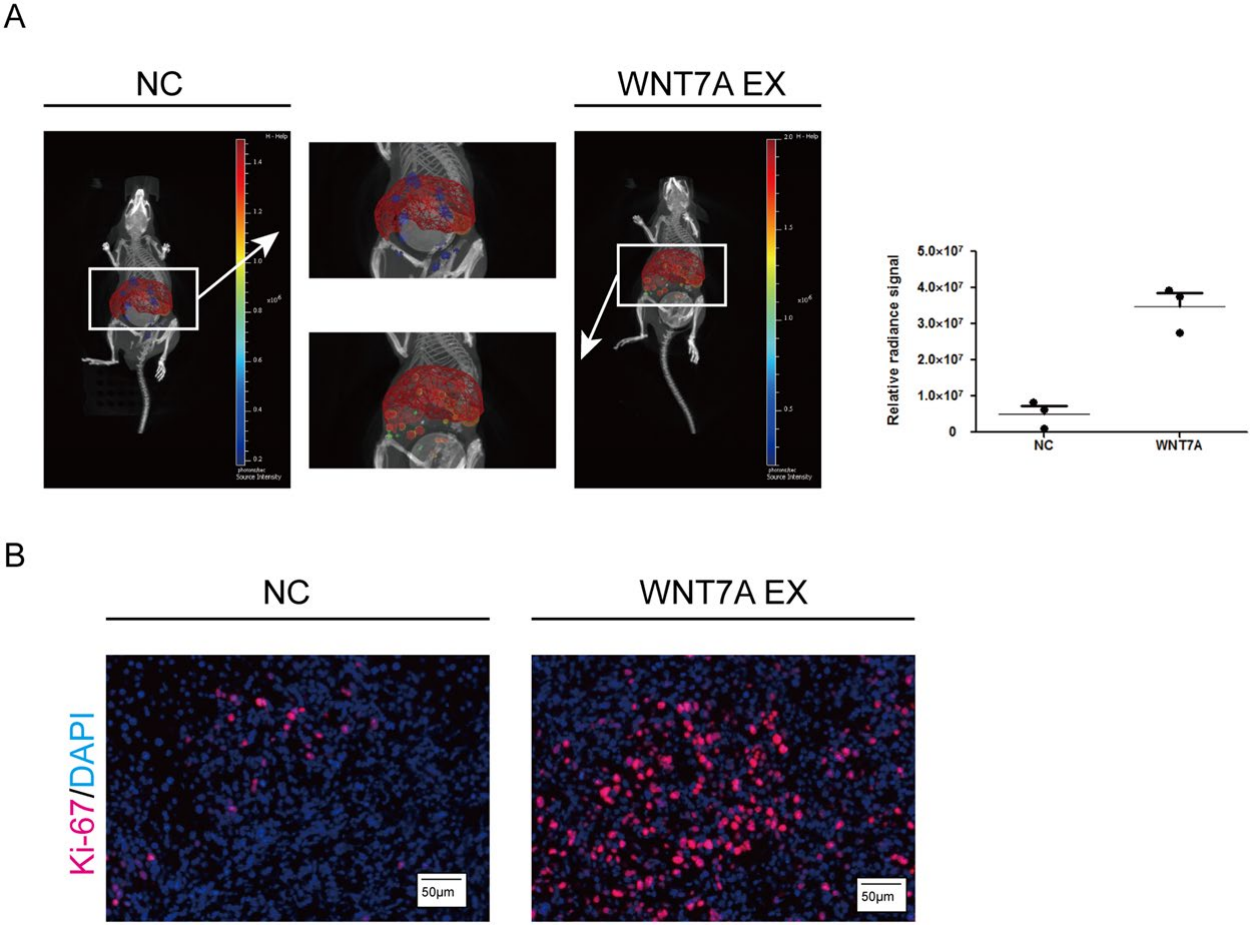
Over-expression of WNT7A promotes the migration capacity of pancreatic cancer cells *in vivo*. (**A**) Orthotopic xenograft were performed in mice by injected with stably transfected cell lines and bioluminescence of metastatic sites are shown. (**B**) Immunofluorescence of orthotopic xenograft liver were performed in which red staining represent Ki-67.

Since the expression of WNT7A is positively correlated with poor prognosis of PDAC cases, we determined to investigate the biological functions of WNT7A in cancer cell lines. In accordance with the observations in tissues, the expression of WNT7A was obviously higher in five pancreatic cancer cell lines than that in nonmalignant hTERT-HPNE cells at both mRNA and protein levels (Fig. 3A). Two PDAC cell lines AsPC-1 and CAPAN-1 with relative high expression level of WNT7A were chosen for knock-down assay using short hairpin RNA (Fig. 3B), PANC1 with relative low expression was selected for over-expression experiments (Fig. 4A). By performing transwell assay, we found that with WNT7A knocked-down, fewer cells migrated to the other side of the chamber compared with control group (Fig. 3C). On the other hand, cells with overexpressed WNT7A showed enhanced capacity to migrate (Fig. 4B). Then we performed wound healing assays, cell migration was significantly decreased in WNT7A knockdown group (Fig. 3D) and enhanced in WNT7A overexpression group (Fig. 4C). To validate our hypothesis *in vivo*, mice PDAC cell line PANC02 with WNT7A up-regulated were established and injected in C57 pancreas as well as wild type PANC02. We found that over-expression of WNT7A significantly facilitated the liver metastasis (Fig. 5A). The immunofluorescence also showed that more proliferated cells were inhabited in liver in WNT7A over-expression group (Fig. 5B). Given the results above, the expression of WNT7A is positively correlated with the migration capacity of PDAC cells.

### WNT7A might promotes the migration capacity of PDAC cell by promoted the EMT process

**Figure 6:**
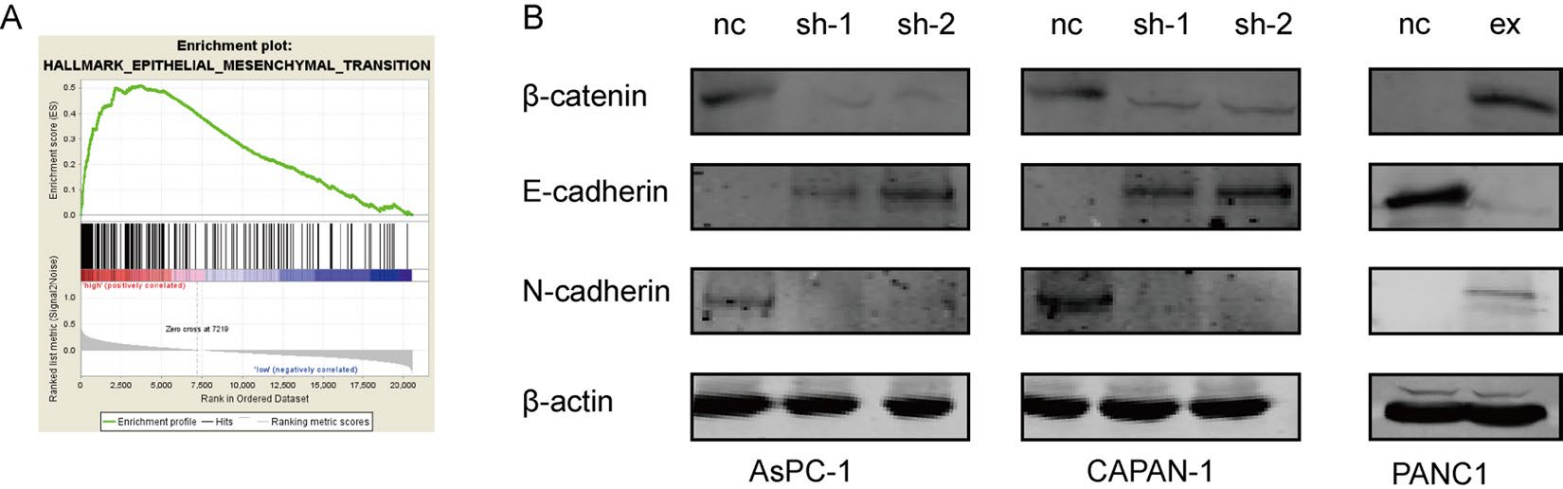
WNT7A promoted the EMT process via activating WNT/β-catenin pathway. (**A**) Gene set enrichment analysis plot based on the gene expression profiles of TCGA samples grouped by expression of WNT7A. (**B**) The expression of β-catenin, E-cadherin and N-cadherin in PDAC cell lines with WNT7A down-regulated and over-expressed are detected by Western blot. The relative expression was normalized to β-actin in cell lines.

To explore the mechanism underlying WNT7A induced migration ability of PDAC cells, we first run the GSEA. The results indicate that the expression of WNT7A may positively correlated with the process of epithelial–mesenchymal transition (EMT) (Fig. 6A). To verify this assumption, we examined the expression of Wnt/β-catenin pathway related proteins including β-catenin, mesenchymal marker N-cadherin and epithelial marker E-cadherin. As shown in Fig. 6B, compared to control group, knockdown of WNT7A in CAPAN-1 and AsPC-1 results in the lower expression of β-catenin and N-cadherin and higher expression of E-cadherin which in contrast is inhibited by over-expression of WNT7A in PANC1 cell lines. These results suggested that WNT7A might enhanced the migration by promoting EMT process.

### The expression of WNT7A is up-regulated in hypoxia condition

**Figure 7:**
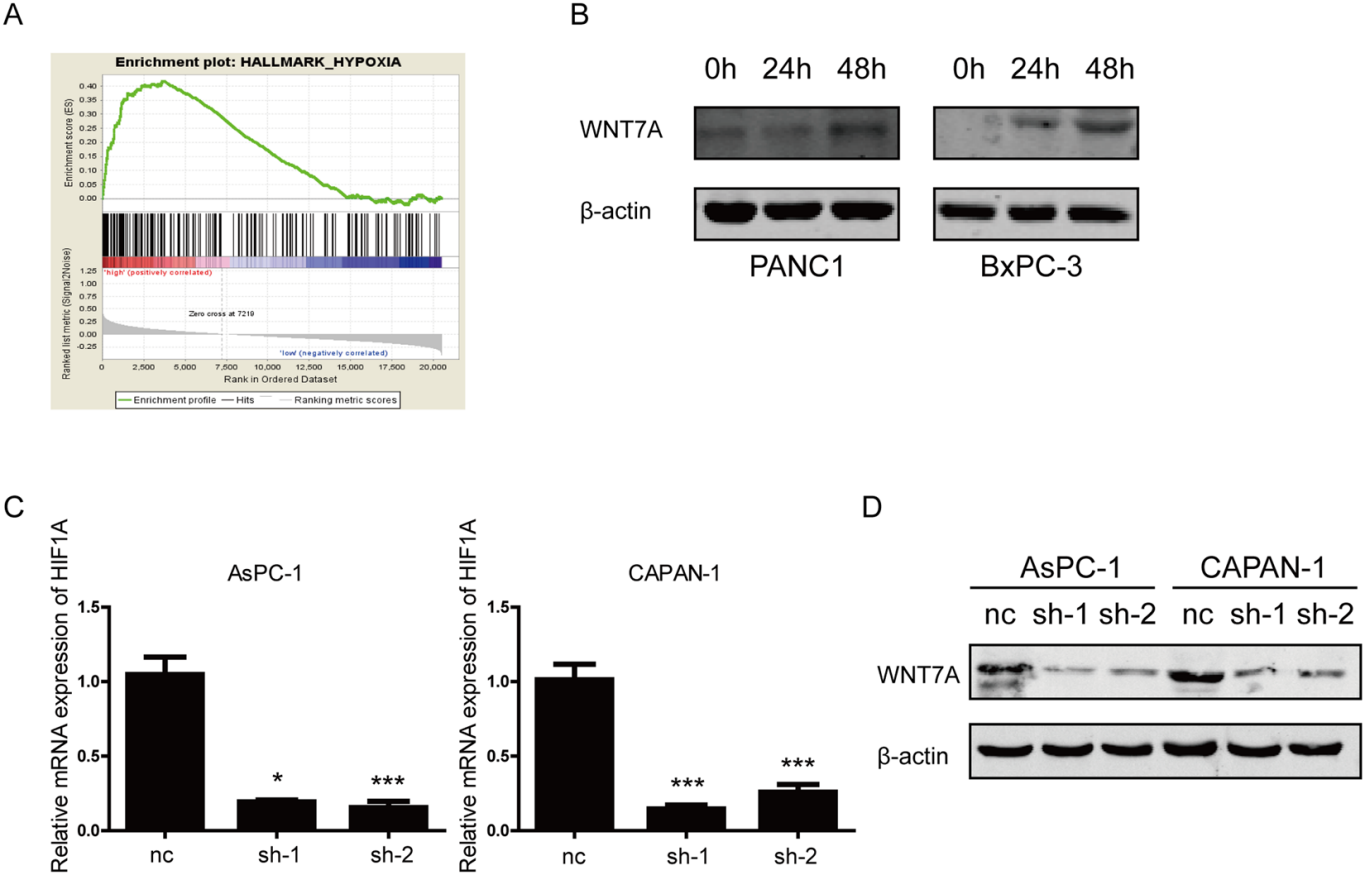
The expression of WNT7A is up-regulated in hypoxia condition. (**A**) GSEA showed the relationship between WNT7A expression and gene set of hallmark hypoxia. (**B**) The protein expression levels of WNT7A in PDAC cell lines cultured in hypoxia for different duration were detected by western blot. (**C**) The knockdown efficiency of HIF1α in PDAC cell lines transfected with interfere plasmid were deteremined by qRT-PCR. (**D**) The effect of of HIF1α knockdown on expression of WNT7A in two PDAC cell cultured in hypoxia condition were detected by Western blot.

Abundant stromal components in PDAC microenvironment is one of the well-known characters of PDAC, which induces a hypoxia condition. Indicated by GSEA analysis (Fig. 7A), we assumed that the expression of WNT7A might be induced by hypoxia. As shown in Fig. 7B, an increased expression pattern of WNT7A occurs at protein level under hypoxia condition. To further explore the mechanism WNT7A up-regulated in hypoxia condition, we interfere the expression of HIF1α in AsPC-1 and CAPAN-1 (Fig. 7C). After cultured in hypoxia condition for 24 hours, the protein expression levels of WNT7A were detected. We found that WNT7A were down-regulated when HIF1α was knocked down in both cell lines, which suggest that overexpression of WNT7A in hypoxia condition may mediated by HIF1α (Fig. 7D).

## Discussion

PDAC is one of the most malignant carcinoma, ranking the fourth leading cause of cancer death in the United States^2^. Many signaling pathways identified to played a pivotal role in the process of tumor initiation and development, among which Wnt signaling is reported to be a crucial determinant of tumor fate for PDAC^15,16^. As one of the WNT ligands, WNT7A contributes to the activation of the canonical WNT/β-catenin via the interaction with Fzd5 and Fzd10. Consist with those results, high expression of WNT7A was found in many cancers including endometrial cancer and ovarian cancer^17,18^. However, it is reported that the expression of WNT7A reduced in cervical cancers, lung cancer and clear cell renal carcinomas^13,19,20^. Since the controversial role of WNT7A, we determined to explore the expression pattern and *in vivo* function of WNT7A in PDAC.

Firstly, we demonstrated WNT7A is overexpressed in PDAC tissue at mRNA level based on the GEO datasets. We performed IHC staining in PDAC TMA, the results confirmed the high expression of WNT7A in tumor tissue at protein level. Then, we found that the expression of WNT7A acted as an independent prognostic factor for PDAC patients, patients with high expression of WNT7A have a dramatically shorter survival time than those with low expression of WNT7A independent of tumor differentiation and lymph node metastasis. The survival analyzes from TCGA datasets demonstrated similar results. Furthermore, we noticed that high expression of WNT7A significantly positive correlated with lymph node metastasis in TMA, which insight the potential link between the WNT7A and tumor metastasis.

To explore the role of WNT7A in tumor metastasis, we performed transwell assays and wound healing assays for cell migration ability. The results suggested that the expression of WNT7A was positively correlated with the migration capacity of PDAC cells. GSEA demonstrated a potential link between WNT7A and EMT process, furthermore, we found that knockdown of WNT7A results in down-regulated expression of N-cadherin and up-regulated expression of E-cadherin. Contrast results was found in WNT7A overexpression group. From these results, we assumed that WNT7A might enhances the migration by inducing EMT process in PDAC cells. It is reported that Wnt/β-catenin signaling pathways contribute to the EMT process^5^. And as one of the secreted proteins of WNT genes family, WNT7A acting as ligand to activate the Wnt/β-catenin pathway^7–9,18^. Based on the reports above, we believed that WNT7A might contribute to the EMT process via activating the Wnt/β-catenin pathway.

Since the expression of WNT7A significantly correlated with patients’ prognosis, the detection of WNT7A in human serum would be greatly valuable. We also explored the underlying mechanism of WNT7A overexpression. The results showed that the expression of WNT7A increased under hypoxia condition at protein level which uncovered a new mechanism of hypoxia microenvironment of PDAC tissue.

In conclusion, the results above demonstrated that hypoxia induced high expression of WNT7A might promote the cell migration via contributing to the epithelial–mesenchymal transition in pancreatic cancer and WNT7A might be a new biomarker for patients’ survival in PDAC.

## Methods

### Patients and Tissue Microarray

Human PDAC tissue microarrays (HPan-Ade180Sur-02) containing 80 cases of tumor and matched adjacent tissue and 20 cases of tumor tissue were purchased from Shanghai Outdo Biotech Inc. All the specimens were obtained from the patients diagnosed with PDAC. The histology and clinical stages were classified according to the seventh edition of the American Joint Committee on Cancer (AJCC) staging system. The follow-up time was calculated from the date of surgery to the date of death, or the last known follow-up. The freshly frozen PDAC tissues and matched adjacent tissues were obtained from Ren Ji Hospital, School of Medicine, Shanghai Jiao Tong University, China. And all patients involved in this study provided written informed consent. The study was approved by the Medical Ethics Committees of Ren Ji Hospital, School of Medicine, Shanghai Jiao Tong University and written consent was obtained from all donors.

### Immunohistochemical and immunofluorescence Staining and evaluation

The human PDAC TMA were deparaffinized and rehydrated. The TMA slides were incubated with 0.3% hydrogen peroxide for 30 minutes and then blocked with 10% Bovine serum albumin (BSA) buffer (Sangon, Shanghai, China) for 20 minutes. The WNT7A antibody (ab217844, abcam, UK) was diluted with optimal proportion, then the TMA slides were incubated overnight with WNT7A antibody solution at 4 °C. After washes, the slides was incubated with HRP (rabbit) second antibody (Thermo Scientific, US) at room temperature for 1 hour. DAB substrate liquid (Gene Tech, Shanghai) were used to positive staining and then counterstained by hematoxylin. All the slides were observed and photographed with a microscope (Carl Zeiss, Germany).

The expression of WNT7A was evaluated according the ratio of positive cells and staining intensity. The intensity was scored as follows: 0, negative; +, weak homogenous cytoplasmic staining; ++, strong staining in <30% of tumor cells; +++, strong staining in >30% of tumor cells. 0 and + were defined as low expression of WNT7A; ++ and +++ as high expression of WNT7A. These scores were determined independently by two pathologists in a blinded manner.

### Cell culture and reagent

Human PDAC cell lines AsPC-1, BxPC-3, CAPAN-1 and PANC1 and normal human pancreatic ductal cell line hTERT-HPNE were all obtained from the Cell Resource Center, Shanghai Institute of Biochemistry and Cell Biology at the Chinese Academy of Sciences (Shanghai, China). All the cells were cultured in appropriate density with American Type Culture Collection (ATCC, Manassas, VA) recommended medium. The medium was supplemented with 10% (v/v) fetal bovine serum (FBS) and 1% antibiotics (100 μg/ml streptomycin and 100 units/ml penicillin). And the cells was cultured in a humidified incubator under 5% CO_2_ condition at 37 °C. To culture cell in hypoxia condition, replacing oxygen with N2 by Heracell 150i CO2 incubator (Thermo Scientific). The concentration of oxygen is monitored by Multi-Gas Detector, model Drager X-am 2000 (Lubeck, Germany). Cells were seeded in 6 cm dishes and grew in hypoxia condition (1% oxygen) for 0 h, 24 h and 48 h. All dishes were seeded and collected to extract RNA and protein at the same time.

### Quantitative Real-time PCR

Trizol reagent (Takara) was used for the total RNA extraction in this study. When the RNA was extracted from the cells, PrimeScript RT-PCR kit (Takara) was used for the reverse transcription. Then Real-time PCR analyses were performed with SYBR Premix Ex Taq (Takara) on a 7500 Real-time PCR system (Applied Biosystems) at the recommended thermal cycling settings. Relative mRNA expression was calculated using the 2^(−ddCT)^ method and normalized to GAPDH mRNA levels. Primer sequences of target genes are listed as follows: WNT7A, forward 5′-CTGTGGCTGCGACAAAGAGAA-3′, reverse 5′-GCCGTGGCACTTACATTCC-3′; GAPDH forward 5′-CTGCCCCCTCTGCTGATG-3′, reverse 5′-TCCACGATACCAAAGTTGTCATG-3′.

### Western blot

Total protein of the cells was extracted by extraction buffer (Beyotime, China) and then the BCA Protein Assay Kit (Pierce Biotechnology) was used to assess the protein concentration. Then lysates were loaded in 10% SDS-PAGE gel and separated after electrophoresis. Then the protein was transferred from the gel to a NC membrane. The membrane then get blocked in 5%BSA for an hour. Primary antibodies WNT7A (ab217844, abcam, UK), c-Myc, β-catenin, N-cadherin, E-cadherin and β-actin (Proteintech, US) were used to probe the specific target protein overnight at 4 °C and then bound with the species-specific secondary antibodies (Proteintech, US) for an hour at room temperature. The concentration of target proteins were determined by Odyssey imaging system (LI-COR Biosciences, Lincoln, NE). All the western blot experiments are repeated 2–3 times.

### Establishment of stable WNT7A knockdown and HIF1α knockdown cell lines

To establish the stable knockdown or over-expressing cell lines, we use lenti-virus packaged by plasmids containing one of two short hairpin RNA (shWnt7a-88, TRCN0000071788 and shWnt7a-91, TRCN0000071791; shHIF1a-1: 5′-GAGGAAGAACUAAAUCCAA-3′; shHIF1a-2: 5′-UGAUACCAACAGUAACCA-A-3′) or WNT7A transcript fragment. Two cell lines AsPC-1 and CAPAN-1 were seed in the 6-well plate at the density of 1 × 10^6^ per well, and cells were infected with 1 × 10^6^ recombinant lentivirus-transducing units with the presence of 5 μg/ml polybrene (Sigma, Shanghai, China). Infected cells were selected using 2 μg/ml puromycin and efficiency was determined by qPCR and Western blot.

### Transwell chamber assays

4 × 10^5^ cells were suspended in serum-free medium and seeded on the upside of the chamber, while medium containing 10% FBS was placed in the lower chamber as the chemo-attractant. After 24 h incubation, the transwell chamber was washed by PBS, fixed and stained with formaldehyde and crystal violet respectively. Cells did not migrate through the filter were removed by cotton stick. The experiments are repeated 3 times independently.

### Wound healing assay

Cells were seeded in the 6-well plates at the density of 1 × 10^6^ per well and form a monolayer. Use 200 μL pipette tip to scratch a cross and then wash for 3 times using PBS. Later, the plates incubate in conditioned medium at 37 °C and photograph at 0, 6, 12 and 24 h. Images were captured and analyzed for the wound closure fraction as a parameter of cell’s migration ability. Three fields were randomly selected from each scratch wound. And the experiments are repeated 3 times independently.

### Orthotopic Xenograft model

C57 mice were kept on a 12-hour day/night cycle with free access to food and water. A total of 2 × 10^6^ PANC02 were injected to the pancreas body of each mice. Metastatic site bioluminescent signals were measured *in vivo* 8 weeks after injection. Then the mice were sacrificed and tumor tissue was collected for immunofluorescence. Experimental procedures involving the animals were authorized by the Animal Ethics Committee of Ren Ji Hospital, School of Medicine, Shanghai Jiao Tong University.

### Statistical analysis

Statistical analysis was performed by SPSS 16.0 (SPSS Inc.; Chicago, IL, USA) and figures were produced by GraphPad Prism 6 (San Diego, CA) software and Adobe Illustrator(ADOBE, USA). *Pearson’s Chi-square* (χ^2^) test was used to analyze the correlations between WNT7A expression and clinicopathologic parameters. We used Kaplan-Meier survival analysis and log-rank test to evaluate the prognostic value of WNT7A expression. Cox regression model was used to examine univariate and multivariate risk factors, then only significantly different variables in univariate analysis including WNT7A expression level, tumor differentiation, N classification were entered into the next multivariate analysis. All statistical tests were two-sided and a *p* < 0.05 was considered statistically significant. Gene set enrichment analysis (GSEA) was performed on the Broad Institute Platform. Samples are collected from The Cancer Genome Atlas (TCGA) and divided into two groups according to the expression level of WNT7A. False discovery rate (FDR) was set at 0.25.

All methods were carried out in accordance with the approved guidelines of School of Medical graduate Shanghai Jiao tong University.

## Electronic supplementary material

Supplementary data

## Data Availability

The datasets generated and analyzed during the current study are available from the corresponding author on reasonable request.
