# Supplementary material for: High expression of WNT7A predicts poor prognosis and promote tumor metastasis in pancreatic ductal adenocarcinoma
Source: Sci Rep. 2018 Oct 25;8:15792. doi: 10.1038/s41598-018-34094-3 (PMC6202314; doi:10.1038/s41598-018-34094-3)
Supplement: Supplementary file 1 — Supplementary data [file 41598_2018_34094_MOESM1_ESM.pdf]

High expression of WNT7A predicts poor prognosis and promote tumor metastasis in pancreatic ductal adenocarcinoma

Dong-jin Wu[1,+], Yong-sheng Jiang[2,+], Rui-zhe He[2,+], Ling-ye Tao[2], Min-wei Yang[2], Xue-liang Fu[2], Jiang-yu Yang[2] & Kun Zhu[3,*]

Supplementary figures

Figure 3A

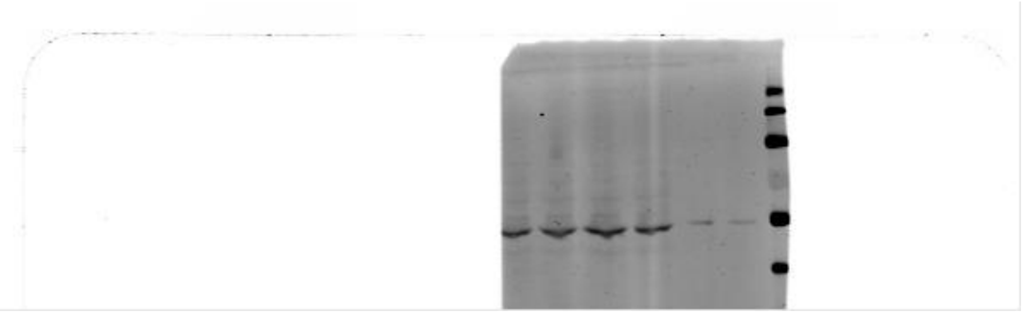

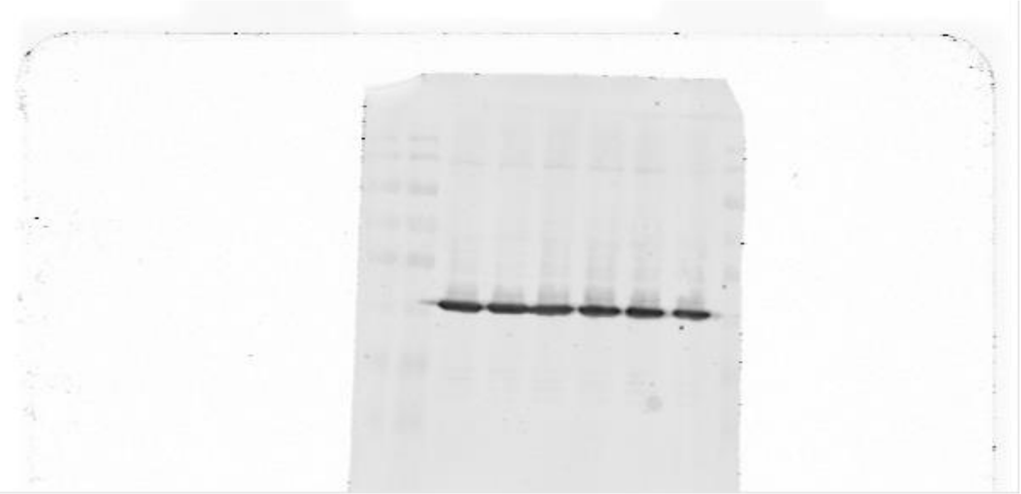

Figure 3B & Figure 4A

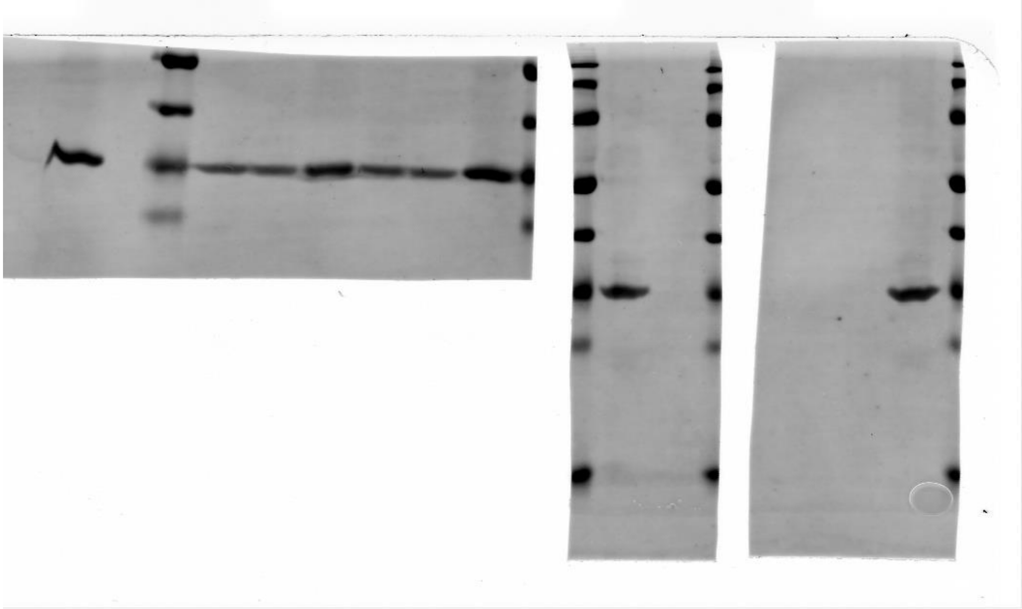

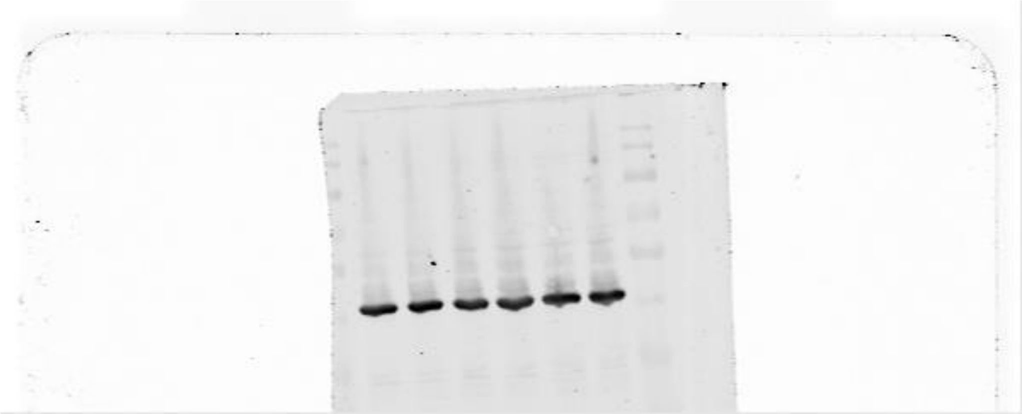

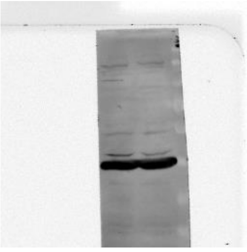

Figure 5B

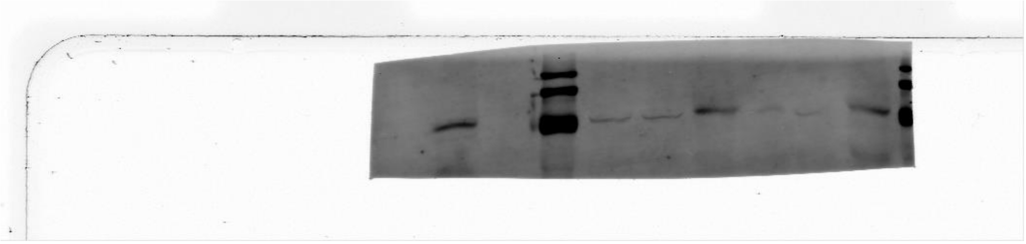

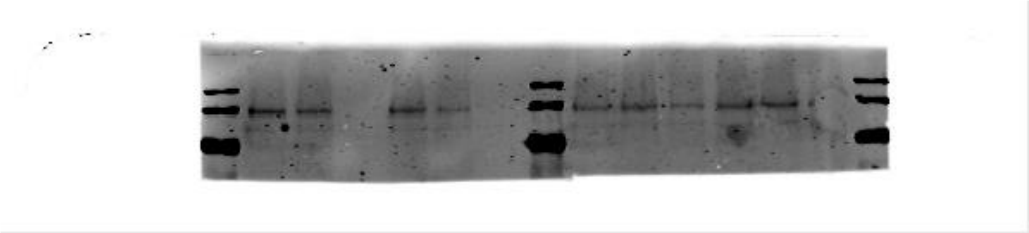

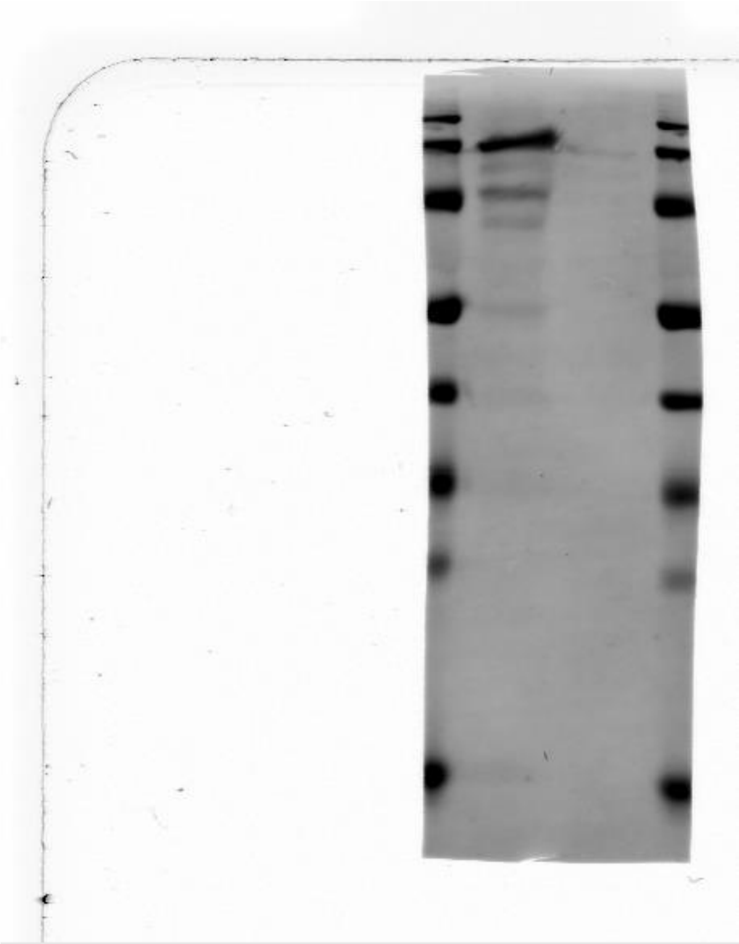

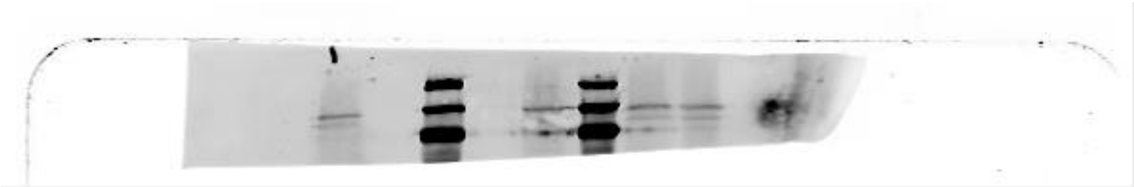

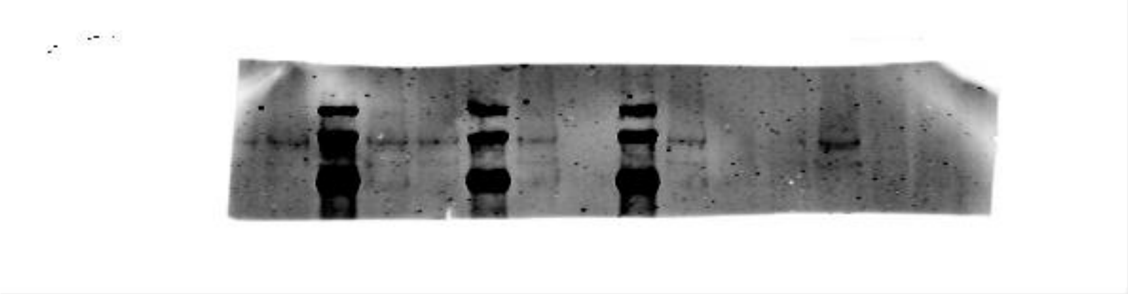

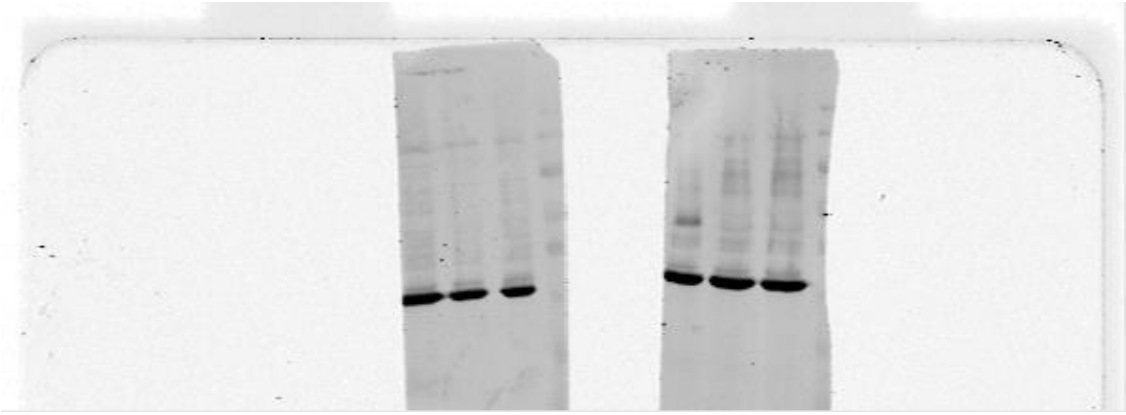

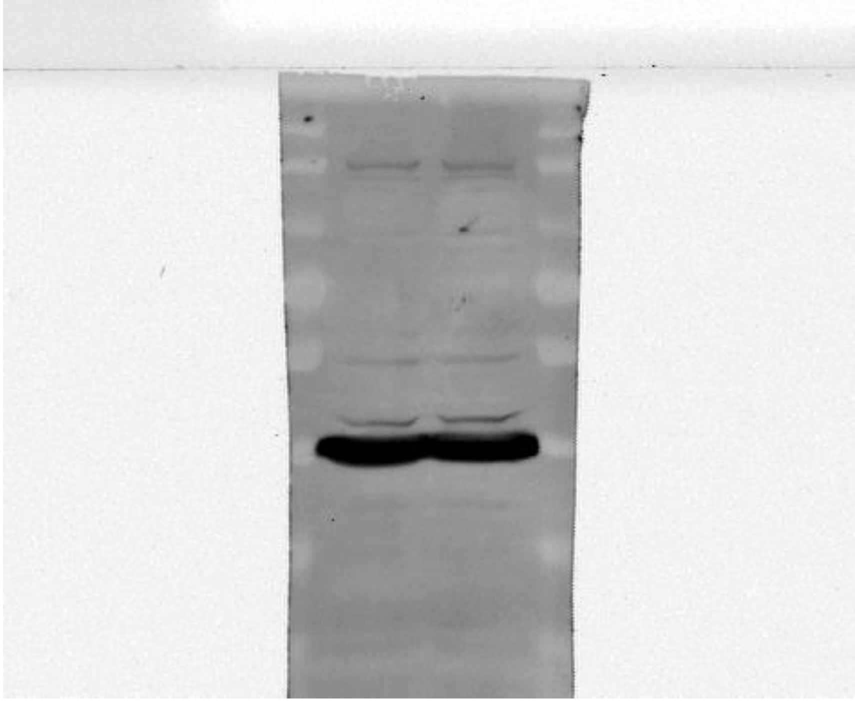

Figure 6B

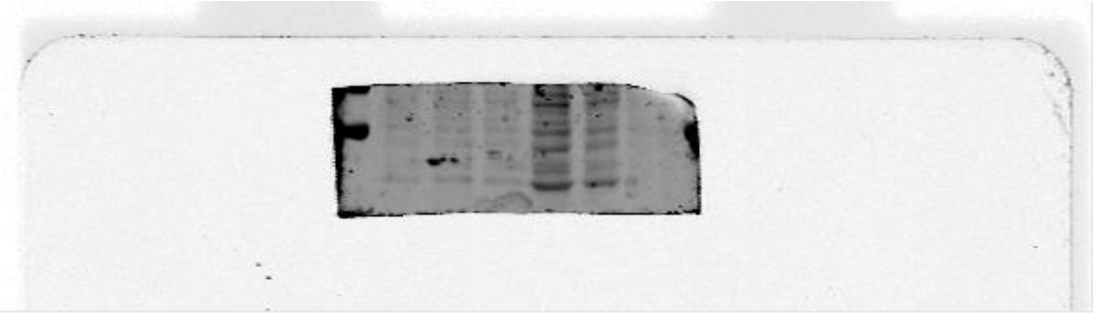

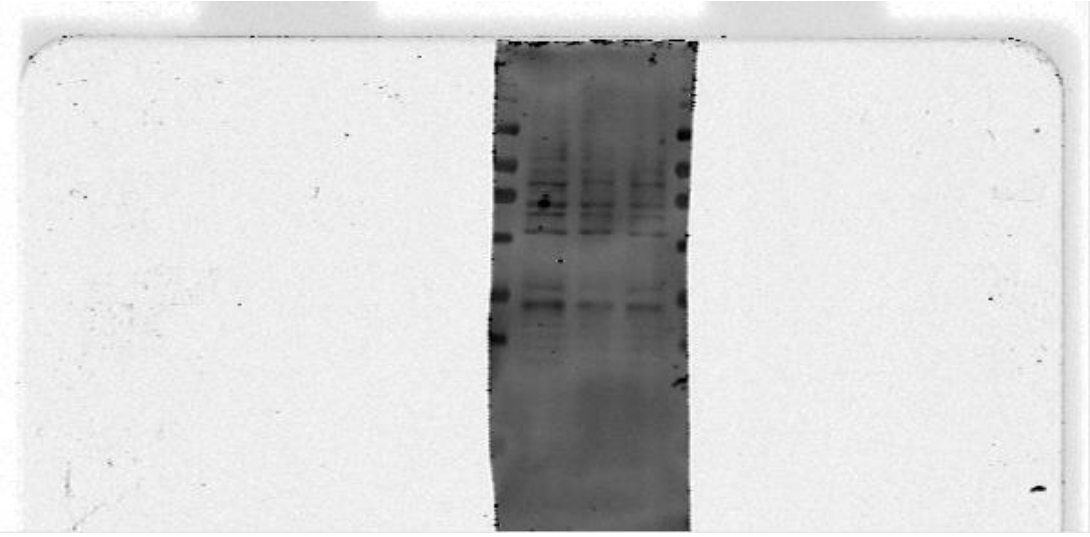

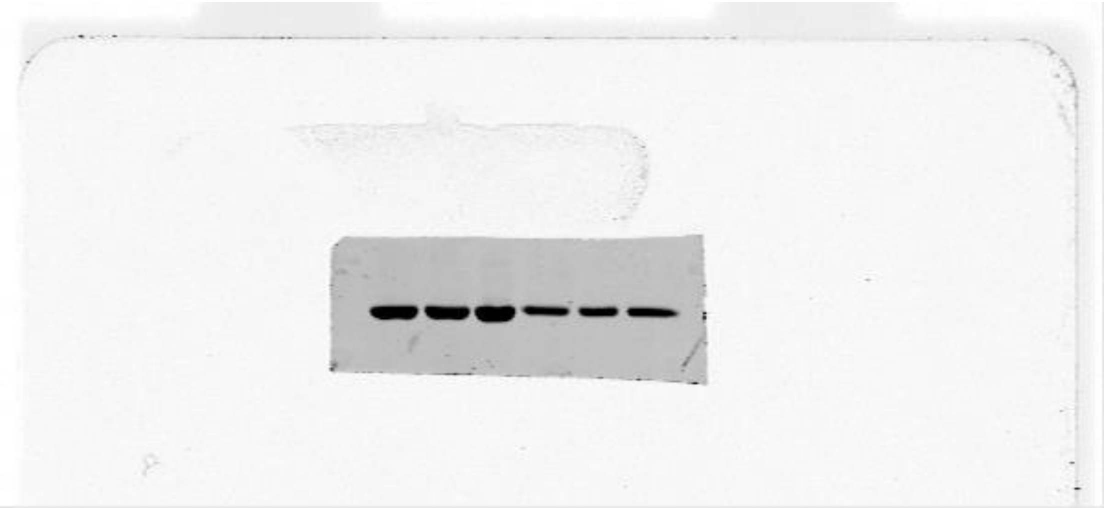

Independent replicates:

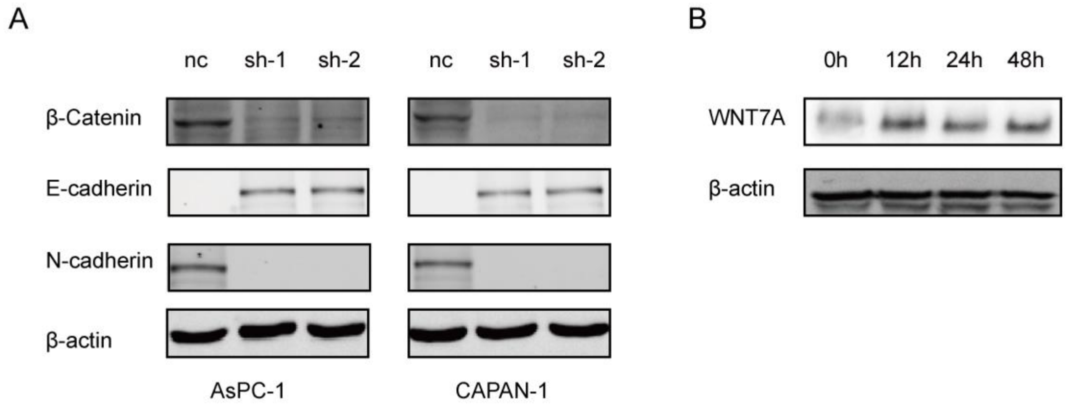

Figure A. The expression of β-catenin, E-cadherin and N-cadherin in PDAC cell lines with WNT7A down-regulated and over-expressed are detected by Western blot. Figure B. The protein expression levels of WNT7A in PDAC cell lines cultured in hypoxia for different duration were detected by western blot.
